# Efficacy of Adding Locoregional Therapy in ATZ/BEV-Treated Patients with Stable HCC

**DOI:** 10.3390/cancers17020185

**Published:** 2025-01-08

**Authors:** Atsushi Hosui, Naoko Hayata, Tomohide Kurahashi, Akane Namiki, Akino Okamoto, Kazuki Aochi, Munehiro Ashida, Takafumi Tanimoto, Hiroki Murai, Kohsaku Ohnishi, Motohiro Hirao, Takuya Yamada, Naoki Hiramatsu

**Affiliations:** Department of Gastroenterology and Hepatology, Osaka Rosai Hospital, 1179-3 Nagasonecho, Kita Ward, Sakai 591-8025, Osaka, Japan; naoko.hayata@gmail.com (N.H.); tkurahashi@osakah.johas.go.jp (T.K.); syu.on.vermillion@gmail.com (A.N.); aknnp.383@gmail.com (A.O.); gierhals.apple@gmail.com (K.A.); mashida1013@osakah.johas.go.jp (M.A.); takafumi1815@gmail.com (T.T.); mhiroki@osakah.johas.go.jp (H.M.); k-ohnishi@osakah.johas.go.jp (K.O.); snowmotty@yahoo.co.jp (M.H.); yamada@osakah.johas.go.jp (T.Y.); hiramatsu@osakah.johas.go.jp (N.H.)

**Keywords:** hepatocellular carcinoma, atezolizumab, bevacizumab, locoregional therapy, trans arterial chemoembolization, ablation, immune boost, stable disease

## Abstract

**Background/Objectives**: Combination therapy with atezolizumab and bevacizumab (ATZ/BEV) is extremely effective and yields a high response rate in patients with hepatocellular carcinoma (HCC). In this study, the efficacy of adding locoregional therapy to ATZ/BEV in patients with stable disease (SD) HCC was investigated. **Methods**: One hundred five HCC patients who were treated with ATZ/BEV or lenvatinib (LEN) as first-line chemotherapy for unresectable HCC were evaluated on the basis of the modified RECIST criteria. SD patients whose initial antitumor effect was achieved received locoregional therapy, and the overall survival (OS) rate was assessed. **Results**: This study included 58 ATZ/BEV-treated participants and 47 LEN-treated participants. Twenty-eight SD patients (ATZ/BEV) and 20 SD patients (LEN) were identified. OS was significantly greater in ATZ/BEV-treated patients who also received locoregional therapy than in those who did not receive this additional therapy (*p* = 0.0343), whereas there was no difference between LEN-treated patients who also received locoregional therapy and those who did not. The locoregional therapy consisted of transcatheter arterial chemoembolization (TACE) and/or radiofrequency ablation (RFA). When assessing the add-on effect of TACE and/or RFA in the SD patients treated with ATZ/BEV, five patients were found to achieve CR. **Conclusions**: The addition of locoregional therapy, such as TACE/RFA, was found to affect SD patients. When a response is limited during ATZ/BEV therapy, it is important to consider the therapeutic option of adding locoregional therapy, as this additional treatment may contribute to improved prognosis via immune modulation.

## Simple Summary

Atezolizumab and bevacizumab were approved as first-line therapies for unresectable or metastatic HCC in 2020, changing the treatment landscape for advanced HCC. However, this systemic chemotherapy is not enough for a cure, and patients with stable disease as the response to this combination therapy are most common. It remains unknown whether this immune therapy should be continued without the addition of any other therapy or with locoregional therapy. Unlike other cancers, locoregional therapies such as transcatheter arterial chemoembolization and radiofrequency ablation are used in existing HCC treatment, and synergistic effects on the immune system are expected with these locoregional therapies. Thus, systemic therapy combined with locoregional treatment has been the focus of recent research. There is an urgent need to identify new treatment strategies that activate the immune system to further improve prognosis.

## 1. Introduction

Sorafenib was approved as the first drug for advanced hepatocellular carcinoma (HCC), on the basis of data from the SHARP study, which was the first study to demonstrate the ability of medical treatment to improve survival in patients with advanced HCC [1,2]. This study set the stage for future drug development in HCC. In the following years, other multitarget tyrosine kinase inhibitors, such as lenvatinib (LEN), which is a first-line therapy, were approved on the basis of positive results in Phase 3 trials [3]. Len has been shown to have a considerably higher rate of objective response and disease control rate in comparison with sorafenib [4]. In 2020, atezolizumab (ATZ), an anti-programmed death ligand 1 (PD-L1) monoclonal antibody, and bevacizumab (BEV), an anti-vascular endothelial growth factor (VEGF) monoclonal antibody, were approved as first-line therapy for unresectable or metastatic HCC, which changed the treatment landscape for advanced HCC [5]. The Phase 3 IMbrave 150 study demonstrated that compared with sorafenib alone, the combination of ATZ and BEV (ATZ/BEV) improved overall survival (OS) and progression-free survival (PFS) [5]. Thus, before the end of 2020, Len was usually used as the first line, and after that, ATZ/BEV was used as the first line. The response-specific analysis of the IMbrave 150 study of ATZ/BEV therapy revealed a difference between responders and nonresponders in terms of prognosis [6,7]. The number of patients with stable disease (SD) in response to ATZ/BEV therapy was greater than those with a complete response (CR) or a partial response (PR) [5]. However, it remains unknown whether this immune therapy should be continued without the addition of locoregional therapy or other therapy. Unlike other cancers, locoregional therapies such as transcatheter arterial chemoembolization (TACE) and radiofrequency ablation (RFA), are used in existing HCC treatment, and synergistic effects on the immune system are expected with these locoregional therapies [8,9]. Thus, systemic therapy combined with locoregional treatment has been the focus of recent research [10,11,12]. Therefore, there is an urgent need to identify new treatment strategies that activate the immune system to further improve the prognosis.

## 2. Materials and Methods

This study evaluated the add-on effects of locoregional therapy in 105 patients with unresectable HCC who were treated with ATZ/BEV or LEN as the 1st-line therapy in our hospital from January 2015 to December 2023.

The ATZ/BEV therapeutic regimen consisted of a combination of 1200 mg atezolizumab plus 15 mg/kg bevacizumab administered intravenously every three weeks until either disease progression (PD) or the onset of unacceptable toxicity. Patients received oral LEN 12 mg/day (for body weight ≥ 60 kg) or 8 mg/day (for body weight < 60 kg). Dose interruptions followed by reductions in LEN-related toxicity (to 8 mg and 4 mg/day, or 4 mg every other day) were permitted.

The inclusion criteria for this retrospective study were as follows: (1) HCC diagnosed on the basis of computed tomography (CT) and magnetic resonance imaging (MRI) or histologically proven in a biopsy specimen; (2) treatment with ATZ/BEV or LEN as the 1st-line systemic therapy for HCC; (3) an Eastern Tumor Cooperative Group Performance Status ≤ 1; and (4) availability of clinical data, including the therapeutic response. The exclusion criteria were as follows: (1) disease complicated with other malignant tumors; (2) history of organ transplantation; and (3) serious complications, including severe heart, lung, kidney, or blood coagulation dysfunction.

Response evaluation: The tumor response, including CR, PR, SD, and progressive disease (PD), was assessed according to the modified Response Evaluation Criteria in Solid Tumors (mRECIST) criteria [13]. Treatment-related adverse events (AEs) were evaluated according to the Common Terminology Criteria for Adverse Events (version 5). The primary endpoints were OS after 1st-line systemic therapy and whether locoregional treatment, as an add-on to ATZ/BEV, resulted in an enhanced therapeutic effect.

TACE therapy: the femoral artery was punctured via the Seldinger technique, and a 5-F catheter and/or a microcatheter were placed in the tumor supply vessels. Then, 5–10 mL of lipiodol and 20–60 mg of epirubicin were mixed into an emulsion and slowly injected.

RFA therapy: RFA was performed via an Arfa (Japan Lifeline Co. Ltd., Tokyo, Japan) RFA system with an adjustable electrode needle. All RFA sessions were performed via a percutaneous approach under ultrasound guidance (LOGIQ E9 XDclear 2.0, GE Healthcare, Chicago, IL, USA).

Ethics statement. This study was approved by the Review Board of Osaka Rosai Hospital (2022-132) and conducted in accordance with the principles of the Declaration of Helsinki. Written informed consent was obtained from all the patients.

Statistical analysis. The two groups were compared with the chi-square test. Normally distributed continuous data are expressed herein as the mean ± standard deviations and were compared with *t*-tests. The OS rate was evaluated via the Cox-proportional hazard model and compared via the log-rank test. Statistical significance was set at a *p* value of <0.05. All the statistical analyses were performed with JMP software (version 12, SAS Institute Japan, Tokyo, Japan).

## 3. Results

### 3.1. Patient Baseline Characteristics

The baseline clinical backgrounds of the 105 patients at the time of ATZ/BEV or LEN induction as the 1st line treatment for unresectable HCC are shown in Table 1. Fifty-eight patients were treated with ATZ/BEV and 47 patients were treated with LEN. Patients in the Len group had longer follow-ups as compared to those in the ATZ/BEV group because Len was approved earlier in the clinical setting. There were no clinically significant differences between the ATZ/BEV- and LEN-treated patients. All patients were in Child-Pugh class A (5/6), 18 (31%) patients were in the ATZ/BEV group and 17 (37%) patients had modified albumin-bilirubin (mALBI) grade 1. The numbers of patients with Barcelona Clinic Liver Cancer (BCLC) stage B and C were 25 and 23 (43% and 40%) in the ATZ/BEV group and 22 and 17 (47% and 36%) in the LEN group, respectively.

### 3.2. Efficacy of Adding Locoregional Therapy to Systemic Chemotherapy for SD Patients Treated with ATZ/BEV or LEN

**Figure 1:**
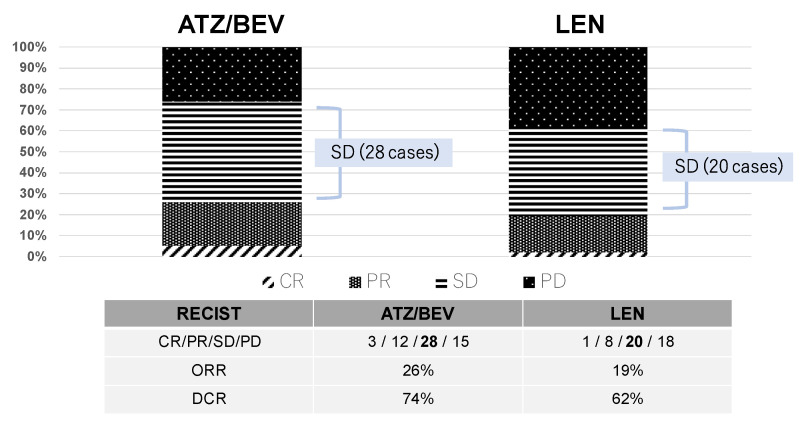
Initial antitumor effect. The effectiveness of these chemotherapies (ATZ/BEV or LEN) was evaluated two months after the initiation of treatment via enhanced CT or MRI. The mRECIST criteria was used in this study. CR: complete response, PR: partial response, SD: stable disease, PD: progressive disease, ORR: overall response rate, DCR: disease control rate.

Two months after the initiation of systemic therapy (ATZ/BEV or LEN), the patients were evaluated by enhanced CT or MRI. Figure 1 shows the effectiveness of these chemotherapies, with DCRs of 74% and 62% and ORRs of 26% and 19% in the ATZ/BEV group and LEN group, respectively. Among these patients, 28 patients in the ATZ/BEV group and 20 patients in the LEN group had SD.

### 3.3. Clinical Courses After Evaluation of the Initial Efficacy in Inducing SD

**Figure 2:**
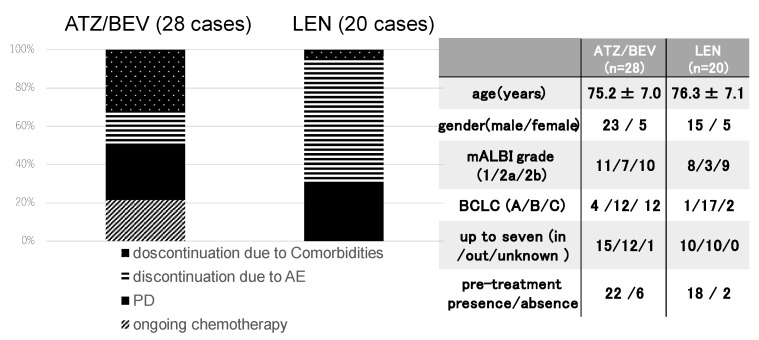
Clinical course after evaluation of the initial efficacy of SD. Left panel: The final status of systemic chemotherapy in SD patients receiving ATZ/BEV therapy (n = 28) and LEN therapy (n = 20). Right panel: Clinical background of the ATZ/BEV group and LEN group used as the first systemic chemotherapy. AE: adverse event, PD: progressive disease, mALBI: modified albumin-bilirubin score, BCLC: Barcelona Clinic Liver Cancer.

The final results of the initial systemic chemotherapy were investigated in the 28 SD patients in the ATZ/BEV group and the 20 SD patients in the LEN group. The clinical background is shown in the right panel of Figure 2. The rate of BCLC-C was significantly greater in the ATZ/BEV group than in the LEN group (43% vs. 10%, *p* = 0.0029), and there were no differences in the clinical background except for tumor burden, between these two groups. The first round of systemic chemotherapy was continued in six patients in the ATZ/BRV group and in no patients in the LEN group. ATZ/BEV therapy was discontinued in 22 patients, and LEN therapy was discontinued in all 20 patients. The main reasons for discontinuation of ATZ/BEV treatment were PD and worsening of comorbidities, whereas most discontinuations of LEN treatment were due to adverse events, such as appetite loss or general fatigue.

### 3.4. Overall Survival Rate with or Without Post-Treatment After Evaluation of the Initial Efficacy of ATZ/BEV in Inducing SD

**Figure 3:**
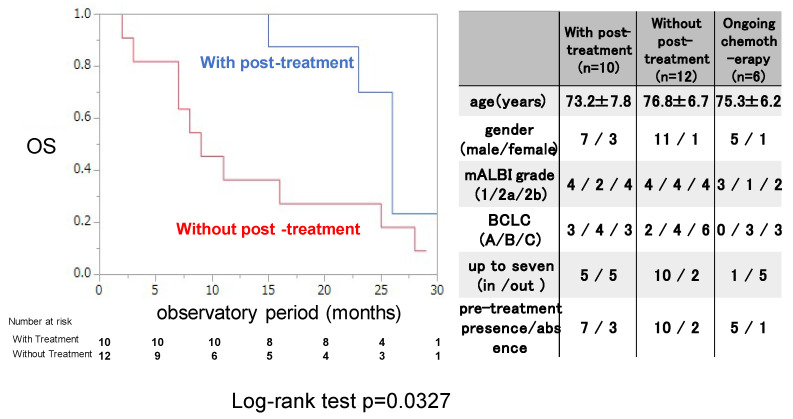
Overall survival with or without post-treatment after evaluation of the initial efficacy of ATZ/BEV in inducing SD. Left panel: Kaplan–Meir curve of SD patients treated with ATZ/BEV therapy with and without post-treatment. The *p*-value was *p* = 0.0327 according to the log-rank test. Right panel: Clinical characteristics of patients receiving ongoing ATZ/BEV therapy with and without post-treatment and AE: adverse event, PD: progressive disease, mALBI: modified albumin-bilirubin score, BCLC: Barcelona Clinic Liver Cancer.

The OS rate was examined for 22 patients who discontinued ATZ/BEV with or without post-treatment. Those with post-treatment were treated with chemotherapy and/or locoregional therapy after 1st regimen of chemotherapy. As shown in the right panel of Figure 3, there were no differences in the clinical background or tumor burden between patients with and without post-treatment with ATZ/BEV. OS was significantly longer in the post-treatment group than in the nontreatment group (MST 25.2 months vs. 10.3 months, *p* = 0.0327).

### 3.5. OS Rate with or Without Locoregional Therapy After Evaluation of the Initial Efficacy of ATZ/BEV in Inducing SD

**Figure 4:**
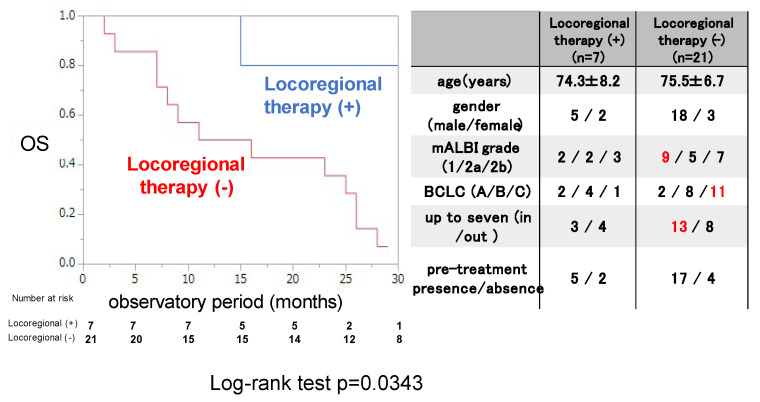
Overall survival in patients with or without locoregional therapy after evaluation of the initial efficacy of ATZ/BEV therapy in inducing SD. Left panel: Kaplan–Meir curve of SD patients treated with ATZ/BEV therapy with and without locoregional therapy. The *p*-value in the log-rank test was 0.0343. Right panel: Clinical background of patients with and without locoregional therapy administered during ATZ/BEV therapy. AE: adverse event, PD: progressive disease, mALBI: modified albumin-bilirubin score, BCLC: Barcelona Clinic Liver Cancer.

Next, the OS rate was examined for 7 patients who were treated with locoregional therapy during and after ATZ/BEV therapy and 21 patients who were not. The clinical background is shown in the right panel of Figure 4. The rate of BCLC-C was significantly lower with locoregional therapy than without it, and there were no differences in clinical background, except for the tumor burden, between these two groups (those without locoregional therapy had a higher proportion of within up-to-7 criteria). The OS rate was significantly greater in patients who received locoregional therapy than in those who did not (MST not reached vs. 15.1 months, *p* = 0.0343).

### 3.6. OS Rate with or Without Locoregional Therapy After Evaluation of the Initial Efficacy of LEN in Inducing SD

**Figure 5:**
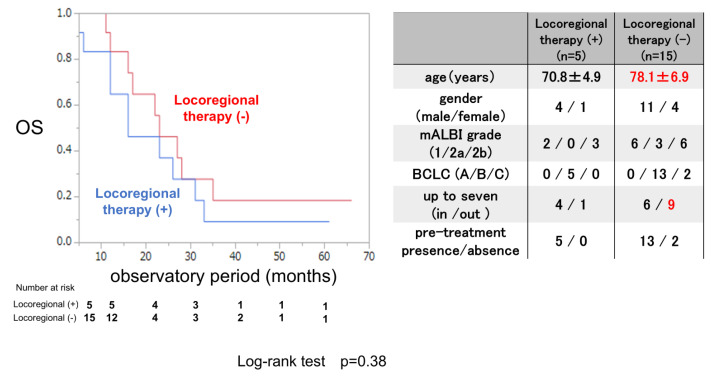
Overall survival in patients treated with or without locoregional therapy after evaluation of the initial efficacy of LEN therapy in inducing SD. Left panel: Kaplan–Meir curve of patients treated with and without locoregional therapy among SD patients receiving LEN therapy. The *p*-value in the log-rank test was 0.38. Right panel: Clinical background of patients with and without locoregional therapy administered during LEN therapy. AE: adverse event, PD: progressive disease, mALBI: modified albumin-bilirubin score, BCLC: Barcelona Clinic Liver Cancer.

The OS rate was examined for 5 patients who were treated with locoregional therapy during and after LEN therapy and 15 patients who were not. The clinical background is shown in the right panel of Figure 5. The mean age was significantly younger in patients who received locoregional therapy than in those who did not receive locoregional therapy, and there were no differences in clinical background except for the mean age between these two groups. The OS rate did not significantly differ between patients who received locoregional therapy and those who did not, not like the ATZ/BEV case.

### 3.7. Case Report of Locoregional Therapy After Evaluation of the Initial Efficacy of ATZ/BEV in Inducing SD

**Figure 6:**
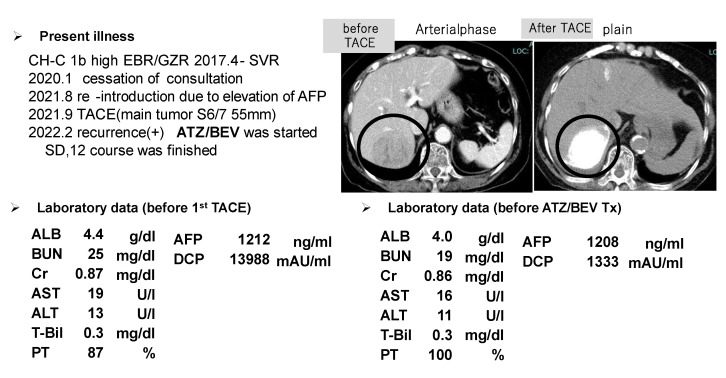
One of the SD patients who received ATZ/BEV therapy and ultimately achieved CR after receiving locoregional therapy. Upper left panel: history of the present illness and HCC therapy. Upper right panel: CT image (left: arterial phase, right plain) before and after TACE (the tumor is marked by a bold circle). Lower panel: laboratory data before TACE (**left**) and before ATZ/BEV therapy (**right**).

The HCC (S6) was found to be approximately 5.5 cm in diameter in an 83-year-old female, and she had no desire for surgery. Her liver function was good with a Child-Pugh score of A, as shown in the left lower panel of Figure 6. TACE therapy was performed for HCC in 2021 (CT image before and after TACE, right upper panel), however, multiple HCC recurrences were observed 5 months after the TACE procedure.

**Figure 7:**
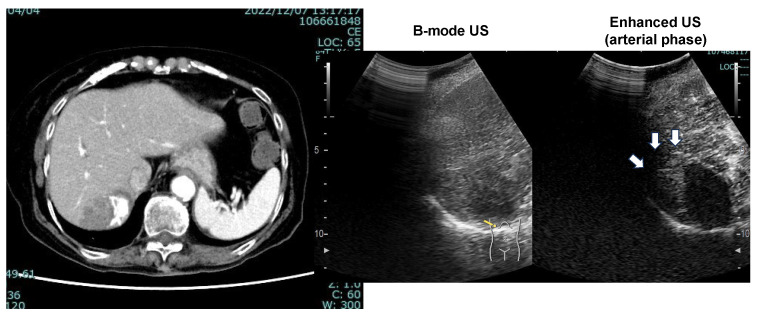
CT image (arterial phase) and enhanced US after 12 courses of ATZ/BEV therapy. Left panel: CT image showing the disappearance of lipiodol and the appearance of arterial flow on the right side of the HCC. Right panel: Contrast-enhanced ultrasound revealing arterial flow in the arterial phase and a defect in the Kupffer phase.

**Figure 8:**
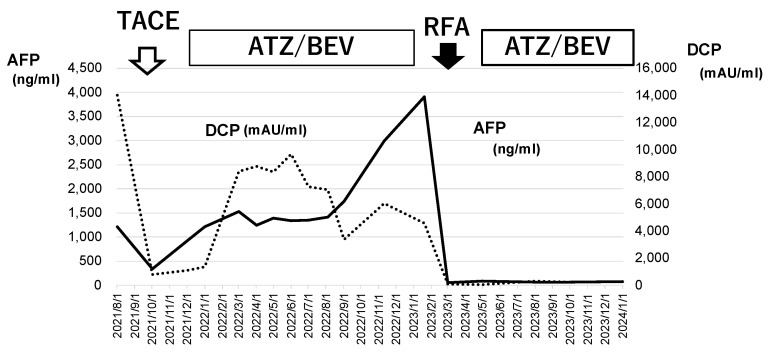
Changes in tumor marker levels during ATZ/BEV chemotherapy and RFA. The values of AFP and DCP before TACE and at the end of ATZ/BEV therapy via the RFA procedure are shown. These tumor markers decreased to normal levels after RFA and remained normal during ATZ/BEV chemotherapy. AFP: alpha-fetoprotein, DCP: Des-γ-carboxy prothrombin, TACE: transcatheter arterial chemoembolization, RFA: radiofrequency ablation.

The patient’s liver function was maintained to Child-Pugh class A, and ATZ/BEV was started. The efficacy of the therapy became evident by the presence of SD after 2 courses of chemotherapy. Subsequently, a total of 12 courses of ATZ/BEV were completed, but contrast US and CT revealed that HCC recurrence was present, however, its status was within the SD category in the modified RECIST (Figure 7 right panel; contrast-enhanced CT arterial phase, left panel; contrast-enhanced US). Finally, RFA was performed on the right side of the S6 HCC (arrow in Figure 7, right panel) and ATZ/BEV combination therapy was restarted after the RFA procedure. A significant reduction in the levels of tumor markers, including AFP and DCP, was observed, and to date, the patient has not had any detectable recurrence of HCC (Figure 8).

## 4. Discussion

The present study indicates that the addition of locoregional therapy is effective for SD patients who have received ATZ/BEV therapy but not for those who have received LEN therapy. Systemic ATZ/BEV therapy is the preferred treatment landscape for HCC, establishing a new standard of care and demonstrating superior long-term efficacy [14]. Combined ATZ/BEV immunotherapy is recommended as the primary treatment for unresectable HCC, according to the guidelines of the BCLC group [15] and the American Society of Hepatology [16] and on the basis of the IMBrave 150 trial findings. However, the CR rate in response to ATZ/BEV therapy was approximately 10%, and the number of patients with SD was greater than those with CR and PR [5]. For carcinomas other than HCC, the only option for SD patients is to continue chemotherapy, which increases the likelihood of PD in the long term.

Recently, many reports have revealed that ATZ/BEV therapy is highly effective and safe and can achieve CR in patients with unresectable HCC, including cases with extrahepatic metastasis, in a real-world clinical setting [17,18,19]. One of the most important issues is obtaining a better prognosis for SD patients who have difficulty achieving a response to therapy with ATZ/BEV alone. The present findings suggest that the combination of ATZ/BEV therapy and locoregional therapy (TACE and/or RFA) may improve the therapeutic effect of ATZ/BEV. This strategy may create an environment in which the therapeutic effect can be maintained and further prolong the prognosis.

This study revealed that LEN therapy was not associated with a better prognosis when locoregional therapy was added to this systemic chemotherapy. In the TACTICS-L trial, LEN-TACE achieved a high response rate and high CR rate and was effective in all subgroups, including the population in whom TACE alone would be less likely to be curative [7]. The researchers concluded that this trial provided encouraging evidence, supporting the efficacy and favorable safety profile of LEN-TACE. What is the difference between our study and the TACTICS-L trial? LEN was administered 14–21 days before the first TACE, stopped 2 days before TACE, and resumed 3 days after TACE in the TACTICS-L study. Vascular normalization (i.e., a reduction in microvascular density, an increase in vessels covered by pericytes, and an increase in perfused vessels) was more pronounced in mice treated with LEN for 4 days [20]. The tumor microenvironment was also more favorable in LEN-treated mice than in control mice, as the LEN-treated mice presented less hypoxic conditions and lower intratumor interstitial pressure. The immune microenvironment was also changed by LEN, which decreased the number of tumor-associated macrophages and increased the number of activated cytotoxic T cells.

In this study, the elimination half-life of LEN was approximately 35 h, and the drug treatment was normally paused for at least 2–3 weeks for procedures that may cause bleeding, such as RFA and TACE. The vascular normalization and favorable tumor microenvironment might have been reversed after drug withdrawal for more than 2 weeks. This difference in the withdrawal period may have affected the prognosis.

Why was adding locoregional therapy effective only in patients receiving ATZ/BEV chemotherapy? ATZ is an immune checkpoint inhibitor that activates the immune system to block the immune escape of cancer cells, allowing autoimmune cytotoxic T cells to efficiently attack cancer cells [21,22]. In addition, anti-VEGF agents promote dendritic cell maturation, normalize the tumor vascular structure, and decrease the number of myeloid-derived suppressor cells (MDSCs) and regulatory T cells [23,24]. The combination therapy of ATZ/BEV thus modifies the tumor microenvironment from an immunosuppressive state to an immunoresponsive state and enhances the antitumor effect of cytotoxic T cells.

The drug penetration provided by TACE also improves the tumor immune microenvironment, including continuous exposure of tumor antigens and enhancement of the efficacy of immune checkpoint inhibitors [25]. TACE also induces local tumor cell death, the presentation of tumor-associated antigens, the migration of dendritic cells into the tumor, and the expression of costimulatory signals, thereby promoting the activation of tumor-specific T cells in the blood circulation and promoting the antitumor effects of cytotoxic T cells. Moreover, in patients undergoing TACE, the possibility of converting the tumor microenvironment from an immunosuppressive state to an immunoresponsive state has also been reported, such as with ATZ/BEV therapy alone [26]. On the basis of the above findings, the addition of TACE to ATZ/BEV therapy may improve the treatment efficacy in unresectable HCC patients.

Ablation, including RFA, increases the number of dendritic cells in the tumor microenvironment of HCC, enhancing antigens and triggering an immune response caused by T-cell activation [27]. In addition to T-cell activation, the most prominent effect is antitumor immunity via the inhibition of bone MDSCs. In this study, the addition of RFA as locoregional therapy improved the OS in patients in whom the effect of ATZ/BEV therapy alone was insufficient.

The elimination half-life of atezolizumab in the human body is 27 days [28], thus, the distribution period is set at 5 months after the last injection, accounting for the elimination period in the blood. In this study, locoregional therapy was performed 21 days after the last ATZ/BEV treatment and restarted approximately 21 days after locoregional therapy. A certain amount of atezolizumab remains in the blood, which is expected to help activate the immune system. This is thought to differ from the case of LEN. Liver function was reported to be maintained during ATZ/BEV therapy but gradually decreased during LEN therapy [29]. This was thought to be the second reason for the better OS in ATZ/BEV, not LEN, with locoregional therapy. Locoregional therapy was repeatedly performed after ATZ/BEV therapy in more cases.

This study has several limitations. First, it was a retrospective investigation, and the sample size was small. Second, locoregional therapies included both TACE and RFA, and this cohort was heterogenous. Moreover, there was a bias between patients with and without locoregional therapy; however, these groups had almost the same clinical background except for the BCLC status. Despite these acknowledged limitations, our study provides further evidence supporting the tolerability and efficacy of the addition of locoregional therapy to ATZ/BEV therapy.

## 5. Conclusions

In conclusion, the combination of ATZ/BEV and locoregional therapy is a safe and effective treatment for patients with unresectable HCC when ATZ/BEV therapy alone is not sufficient. When the response is limited during ATZ/BEV therapy, it is important to consider the therapeutic option of adding locoregional therapy, as this additional treatment may contribute to improved prognosis via immune modulation, with tolerable adverse reactions.

## Figures and Tables

**Table 1:** Clinical background of 105 patients who were treated with ATZ/BEV combination therapy or LEN therapy as the 1st line treatment.

	ATZ/BEV (n = 58)	LEN (n = 47)
age (years)	75.4 ± 8.4	75.5 ± 7.9
gender (male/female)	51/7	30/17
etiology (HCV/HBV/Alc/MASH/AIH/PBC/unknown)	14/7/15/11/2/1/8	19/5/6/10/4/0/3
mALBI grade (1/2a/2b)	18/23/17	2017/9/21
BCLC (A/B/C)	10/25/23	8/22/17
up to seven (in/out/unknown)	28/23/7	14/29/4
the number of intrahepatic tumors(≤5/5</unknown)	33/18/7	23/20/4
maximum tumor size (≤30 mm/30 mm</unknown)	26/25/7	28/15/4
extrahepatic tumor (lymph node/lung/bone, soft tissue/portal vein/others)	5/6/3/3/1	2/5/2/7/4
AFP (ng/dL)	22.9 ± 7652	72.1 ± 28511
PIVKA II (mAU/mL)	238 ± 20403	941 ± 12449
pre-treatment present/absence	45/13	39/8

## Data Availability

No new data were created or analyzed in this study. Data sharing is not applicable to this article.
